# Data management architecture, University Hospital Haiti

**DOI:** 10.3402/gha.v9.32195

**Published:** 2016-06-07

**Authors:** Yuri Zelenski, Dieter Eisenmann

**Affiliations:** 1 Faculty of Medicine and Pharmacy, State University of Port-au-Prince, Port-au-Prince, Haiti; 2 Foundation in Support of the Ophthalmology Department, Hospital Albert Schweitzer Haiti, Chur, Switzerland; 3 DGZ International, Ottawa, Canada, Email: yuri.zelenski@gmail.com; 4 Kantonsspital Graubünden, Chur, Schweiz, Email: dr.d.eisenmann@gmail.com

**Figure:**
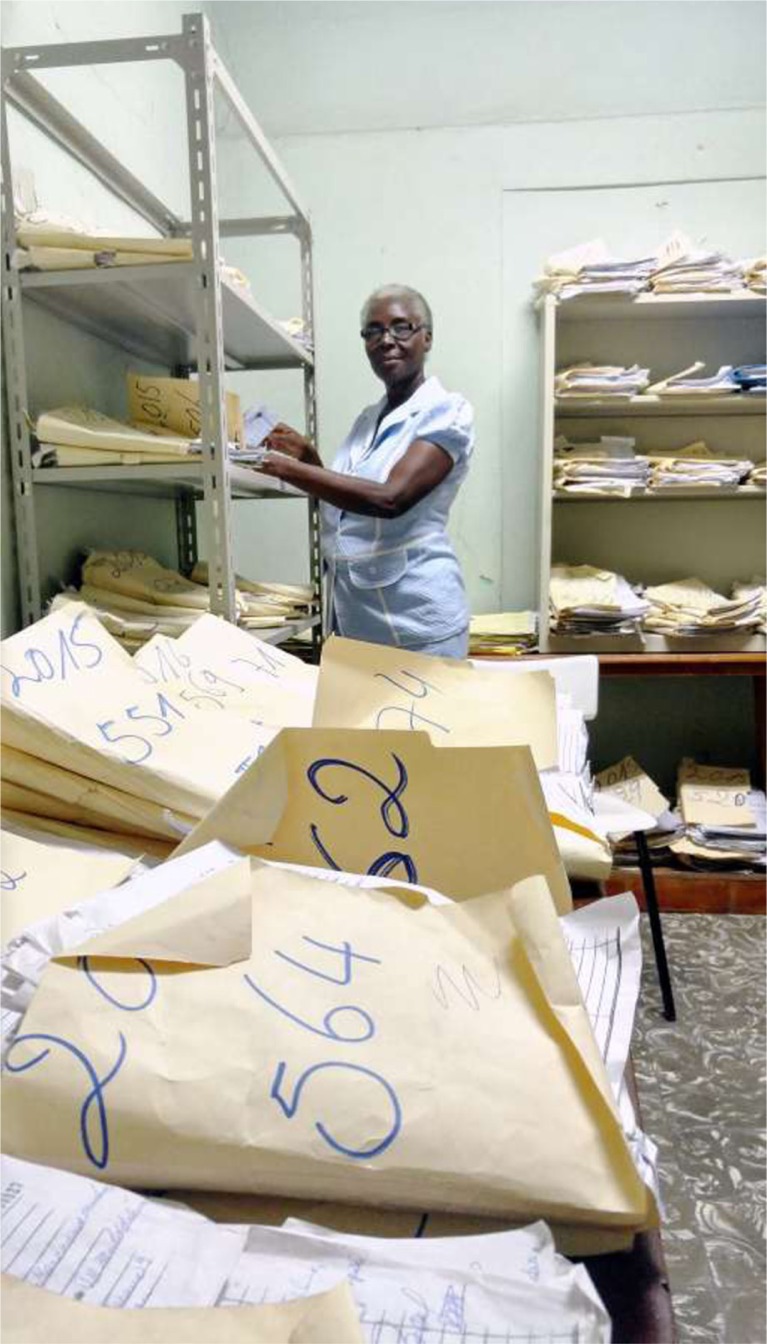


Archives administrator Mrs. Yolette Délius works with paper files for information provision in the Archives of the Ophthalmology Department of State University Hospital Haiti on January 26, 2016. The existing data management architecture is unable to provide reliable indicators for data-driven management and policy due to the lack of financing, digital technologies, adequate human resources, and technical expertise (1). Digital transformation requires different skills, equipment, regulation, policy, internet access, electricity, and maintenance (1–3). This capture is important as it snapshots nondigitalized data process business environment in the settings of the leading national public health institution and provides arguments to inform decision makers about a significant gap between digital technology and institution as well as contribute to generating institutional changes at the country level.

*Yuri Zelenski* Faculty of Medicine and Pharmacy State University of Port-au-Prince Port-au-Prince, Haiti Foundation in Support of the Ophthalmology Department Hospital Albert Schweitzer Haiti Chur, Switzerland DGZ International Ottawa, Canada Email: yuri.zelenski@gmail.com *Dieter Eisenmann* Foundation in Support of the Ophthalmology Department Hospital Albert Schweitzer Haiti Chur, Switzerland Faculty of Medicine and Pharmacy State University of Port-au-Prince Port-au-Prince, Haiti Kantonsspital Graubünden Chur, Schweiz Email: dr.d.eisenmann@gmail.com
